# A New Hypothesis on Anxiety, Sleep Insufficiency, and Viral Infections; Reciprocal Links to Consider in Today's “World vs. COVID-19” Endeavors

**DOI:** 10.3389/fpsyt.2020.585893

**Published:** 2020-11-05

**Authors:** Mohammad Nami, Samrad Mehrabi, Ali-Mohammad Kamali, Milad Kazemiha, Jose Carvalho, Sabri Derman, Johant Lakey-Betia, Velmarini Vasquez, Rao Kosagisharaf

**Affiliations:** ^1^Department of Neuroscience, School of Advanced Medical Sciences and Technologies, Shiraz University of Medical Sciences, Shiraz, Iran; ^2^Dana Brain Health Institute, Iranian Neuroscience Society-Fars Chapter, Shiraz, Iran; ^3^Academy of Health, Senses Cultural Foundation, Sacramento, CA, United States; ^4^Neuroscience Center, Instituto de Investigaciones Científicas y Servicios de Alta Tecnología (INDICASAT AIP), Panama City, Panama; ^5^Sleep Disorders Laboratory, Namazi Hospital, Shiraz University of Medical Sciences, Shiraz, Iran; ^6^Division of Pulmonology, Department of Internal Medicine, School of Medicine, Shiraz University of Medical Sciences, Shiraz, Iran; ^7^High Performance Brain, Rotterdam, Netherlands; ^8^Sleep Disorders Unit, American Hospital, Koc Foundation, Istanbul, Turkey; ^9^Centre for Biodiversity and Drug Discovery, Instituto de Investigaciones Científicas y Servicios de Alta Tecnología (INDICASAT AIP), Panama City, Panama

**Keywords:** sleep disorders, anxiety, innate immunity, viral infections, COVID-19, nutraceutical

## Abstract

In today's ever-growing concerns about the coronavirus disease (COVID-19) pandemic, many experience sleep insufficiencies, such as difficulty falling or staying asleep, sleep-related behavioral symptoms, and out-of-phase circadian rhythmicity despite the lack of history of earlier such symptoms. Meanwhile, the disruption in sleep bioparameters is experienced more in people with a history of sleep disorders. The behavioral sleep disorders in the current situations are prevalent given the today's amount of anxiety everyone is feeling about COVID-19. On the other hand, evidences indicated that the cross-link between impaired sleep efficiency and disrupted innate immunity makes people susceptible to viral infections. The present brief review highlights the links between psychosocial stress, sleep insufficiency, and susceptibility to viral infections in relevance to COVID-19 situation. The stress management measures, including addressing sleep-related disorders and sleep hygiene, will have a notable impact by harnessing immune response and thus reducing the susceptibility to viral infections.

## Introduction

Sleep health has been addressed as a key pillar for overall health in humans (1–3). Nonetheless, owing to the noticeable anxiety related to the health-related crisis of coronavirus disease (COVID-19), many people are experiencing sleeping difficulties (3). People tend to report disrupted or out-of-phase circadian rhythmicity, as well as aggravated underlying sleep related predicaments secondary to the pandemic-related anxiety (3).

Given the fact that on March 22, 2020 was World Sleep Day (WSD), many academic platforms introduced tools and provided advice to assist people improve their sleep despite the current stressful situation. Sleep hygiene measure, stress management tools, and addressing sleep-related disorders were the highlights of the advice provided in the media during the WSD (4).

The anxiety will influence and change the sleep pattern and duration (4). It is important to note that both short and long sleep durations are important for human global health (4). The resultant little stage 3 sleep and too long REM sleep affect the hormonal responses (5–7).

Indeed, evidences have postulated that patients with sleep disorders, such as restless legs syndrome, sleep-phase disorders, sleep-related movement disorders, insomnia, hypersomnia, parasomnias, and sleep-disordered breathing [i.e., obstructive sleep apnea–hypopnea syndrome (OSAHS)] are not only exposed to cognitive and affective predicaments, namely, anxiety and dysregulated mood, but also have impaired immunity (8–13). In other words, proper sleep indices are known to positively influence cognitive fitness, affective health, emotions, immune system's aptitude, metabolism, and appetite (3, 14).

There is considerable evidence explaining the potential links between sleep disorders, anxiety, and impaired immunity (i.e., susceptibility to viral infections) (15–19). A systematic literature search in PubMed, Scopus, and Google Scholar databases (1980–2020) was conducted using the combination of our keywords to isolate relevant original research articles that investigated the cross-link between sleep, anxiety, and immunity. A multidisciplinary expert panel decided on selecting the most relevant articles after two webinar sessions to discuss summary and conclusions.

In addition to the chronic anxiety and immune dysfunction in relation to impaired sleep integrity and efficiency, limited studies indicated the possible consequences of home quarantine on anxiety and sleep disturbances, etc. (20–22). Taking these considerations into account, the present review aims to create awareness on sleep health and mental hygiene and immunity against viral infections, namely, COVID-19. In addition, multidisciplinary initiatives encompassing medical, psychobehavioral, and sleep health clinical service provision would need to be considered to further help with today's burden of the COVID-19 pandemic. These are current and future direction studies.

Given the above, the present brief review attempts to provide a new hypothesis on multifaceted links between the anxiety, sleep disturbance, and innate immunity against viral infections in the COVID-19 era.

## Anxiety and Innate Immunity (Susceptibility to Viral Infections)

It has been indicated that psychosocial stress results in dysregulated endocrinoimmunological responses leading to increased circulating cortisol level, as well as steroid insensitivity. And this would, in turn, leads to declined circulating neural growth factor and brain-derived neurotrophic factor (BDNF), whereas proinflammatory mediators surge on the opposite (11, 23). The resulting neuroinflammatory response induces a cytokine reaction and subsequently activates the “cytokine–hypothalamic–pituitary–adrenal–cytokine” axis prompting a dysregulated immune response (24, 25). The anxiety-induced neuroinflammatory response in the brain causes mononuclear cell infiltration and up-regulates interferon-α receptors, which in turn triggers a continued neuroimmunological reaction. Despite the above, the role of neuroinflammatory responses in viral infections is not well-established in the field and need to be further elaborated (24, 25).

Earlier studies have indicated the disruption in several immunity variables in animal models. For example, the anxiety-induced impairments in immune response was restricted to not only the cellular immunity (i.e., total number of lymphocytes, granulocytes, monocytes, helper, and cytotoxic T cells, as well as natural killer cells), but also humoral immunity measures, such as circulating immunoglobulins A, G, and E (10). Such results have proposed that anxious animal could be more susceptible to infections and inflammation, especially when they were exposed to a chronic state of anxiety (10, 14, 26).

Studies related to the effects of anxiety on innate immunity are relatively sparse. Evidences have substantiated that while the chronic state of anxiety appears to notably impair the innate immune function, acute and subclinical stress may lead to augmented immunity (27). This might be a transient compensatory reaction only before the immune response is down-regulated. From the psychoimmunological viewpoint, we demonstrated in an earlier report that practicing mindfulness exercise and mantra meditation to counteract anxiety could reinforce some humeral immune competency measures (27). Nevertheless, the relation between emotion regulation and immunity needs to be further investigated based on which the effects of mental health treatments either through pharmacotherapy or psychobehavioral approaches on immunity would possibly be better explained in future studies (8, 28).

## Sleep Disorders and Anxiety

Sleep is known to be a pillar of both physical and mental health (3). It is well-documented that proper sleep can optimize one's quality of health, productivity, and functional capacity (3). On the other hand, the personal and societal burdens of sleep disorder are significantly important in human health. When sleep insufficiency becomes an issue, people feel consequences, such as impaired interpersonal and social relationships, decreased academic and work-related functions, and also impeded cognitive and behavioral aptitude partly reflected on one's ability in decision-making (2, 3). We presume what, in turn, may cause anxiety-related disorders?

There exists considerable evidence regarding the role of exercise as a positive influencer and “improver” of sleep behavior (29). Furthermore, studies have substantiated exercise-induced reduction in stress-related anxiety (29, 30). Nevertheless, with today's special COVID-19 circumstances, where most people are quarantined at their home and follow a sedentary lifestyle, insufficient physical activity may be perhaps a potential contributor for the rise of anxiety and sleep disorders (31).

With regard to COVID-19 related anxiety, which surrounds almost everyone these days, individuals' sleep patterns are potentially disrupted (31, 32). People are constantly following up with unpleasant news, worrisome updates, and distressing limitations broadcasted in the media. We hypothesize that all the psychobehavioral burden of such an anxiety and the increased screen time *per se* may affect the brain circadian rhythmicity causing individuals' sleep out-of-phase. The delayed sleep phase disorder secondary to browsing social media late at night can result in “social jet lag.” The phenomenon is expected to leave negative impacts on cognition, cognitive–emotion regulation, behavioral organization, and mood state, as well as bodily responses, such as impaired endocrine–immunological functions (33, 34).

It should be noted that people with preexisting sleep-related disorders are more likely to experience today's COVID-19 anxiety burden. This may be partly due to disrupted sleep macrostructure (i.e., appropriate proportions of slow-wave sleep and rapid eye movement sleep) that deprives the brain and musculoskeletal system to undergo tranquility and restoration (3). When such individuals, especially the older adults and those with preexisting conditions, learn about their vulnerability to COVID-19, the vicious cycle of poor sleep and impaired functionality gets refueled by another potential factor, i.e., “the anxiety” (20, 35).

Many patients with OSAHS already may have comorbidities, such as diabetes, coronary artery disease, lung disease, or chronic obstructive pulmonary dysfunction (12, 36, 37). The fact that these individuals who already have sleep disorders, such as OSAHS plus an underlying disease are at higher risk for COVID-19 (38) thus risks them in chronic anxiety state, which *per se* can further impede their sleep efficiency (39).

For those cases with OSAHS who receive continuous positive airway pressure (CPAP) therapy, device hygiene and safety measures, such as proper titration to ensure adjusted flow are important. Hyperaeration by CPAP would increase the risk of aspirating saliva deep into the lung while asleep, which would enhance the risk of lung infection (12, 40). Patients who receive therapy for sleep disorders need to stay further vigilant about the possible symptoms of COVID-19 and contact their health care professional preferably for teletherapy.

From the chronobiology standpoint, prolonged home stays may place undue strain on the body's circadian timing system, whereby straightforward advice to alleviate the issue needs to be provided where applicable. In the same vein, a recent report has brainstormed the impact of COVID-19 and physical distancing as risk for circadian rhythm dysregulation and needs further investigation (41).

However, healthy individuals with no prior history of sleep disorders may also experience disrupted sleep in the current situation. The stressful condition positions individuals in varying levels of chronic anxiety, which can subsequently affect their sleep efficiency. Furthermore, the financial and social burden of the issue adds to the mental health burden of the disease or condition. To counteract some of these challenges, referrals are mainly advised to adhere to sleep hygiene measures and practice mindfulness, meditation, and relaxation exercises (33, 42, 43).

Several studies have supported the contribution of routine duties for sleep quality. Because many people are breaking their routines in today's situation because of quarantine and impaired social contacts, sociobehavioral programs need to be planned to help the community (44–46).

## Sleep and Innate Immunity (Susceptibility to Viral Infections)

The modulatory effects of sleep on immune response has been well-articulated in the literature (44, 45). Inefficient sleep is thus known to downscale the immune system's competence, resulting in one's vulnerability to infectious diseases. Some studies have demonstrated that decreased total sleep time is correlated with prolonged suffering from common cold (15, 45).

With the significance of today's viral pandemic, we tend to focus on the links between sleep insufficiency and some viral infections including COVID-19. In order to explain the underpinning mechanisms through which sleep insufficiency increases the risk of viral infections, sleep deprivation animal models have brought about evidence in an earlier study (26). Such evidence has revolved around behavioral, cognitive, immunological, and neurochemical alterations after sleep deprivation.

Sleep loss is shown not only to increase tumor necrosis factor-α (TNF-α) but also, in turn, causes inflammatory consequences in a rat model (26). Moreover, partial sleep restriction has also been linked with unfavorable immune response reactions, such as diminished lymphocytes, mitogenic proliferation, down-regulated expression of human leukocyte antigen-DR isotype, and disproportionately altered helper and cytotoxic T cells, as well as natural killer cells. Such a sleep-related impairment in immune response against viral pathogens would expectedly make individuals with sleep disorder more vulnerable to immune dysfunction (46).

**Figure 1:**
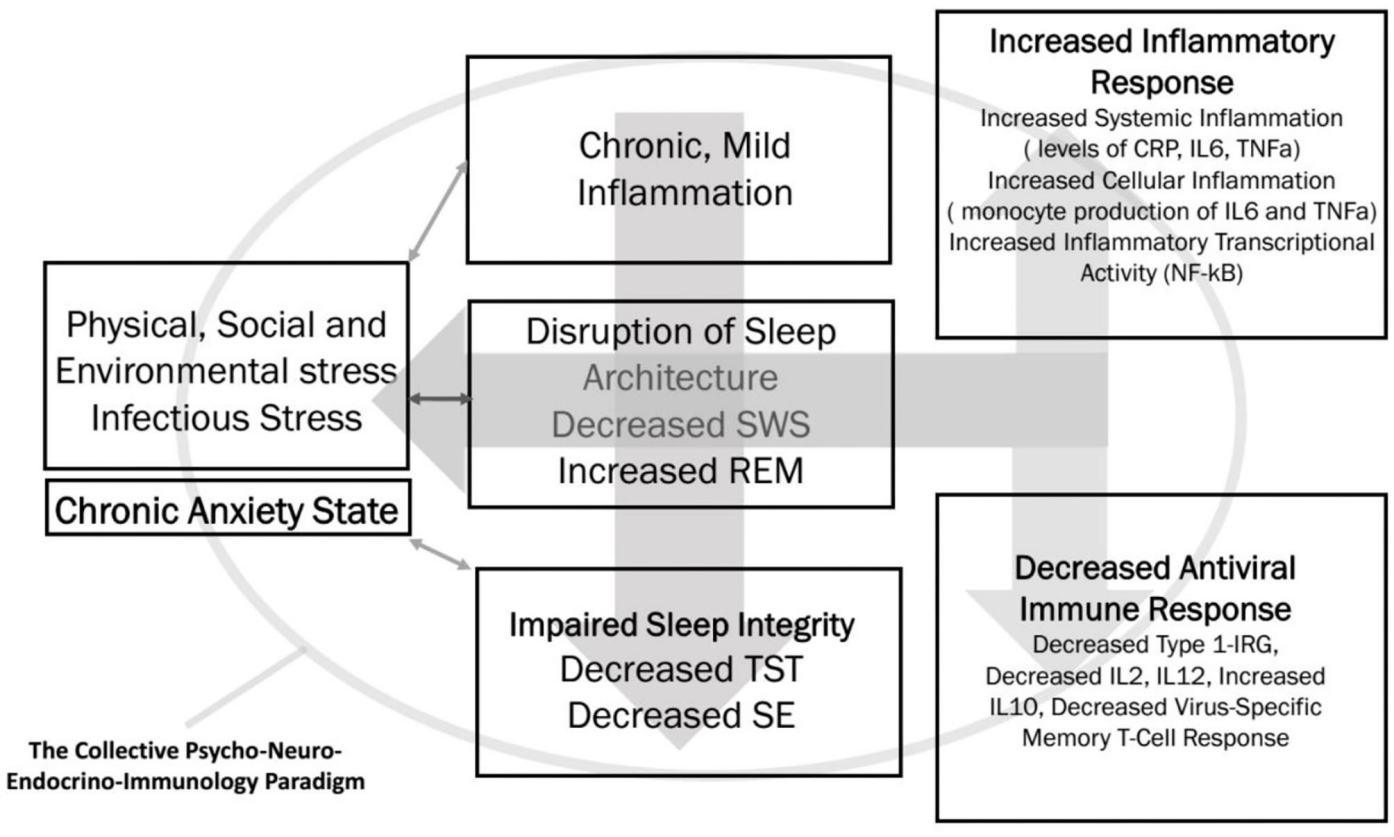
The trilateral interface of chronic anxiety, sleep efficiency, and innate immunity is summarized. SWS, slow wave sleep; REM, rapid eye movement; TST, total sleep time; SE, sleep efficiency; CRP, C-reactive protein; IL, interleukin; TNF-α, tumor necrosis factor-α; NF-κB, nuclear factor κB; IRG, interferon response gene.

As stated earlier, there seem to be multifaceted links between the pandemic-induced anxiety, impaired sleep efficiency indices, and vulnerability to viral infections, which potentially include COVID-19. There are some polysomnographic studies and immunological assays in patients with various types of viral infection. We hypothesize the trilateral links among anxiety, sleep, and innate immunity (20, 24, 32, 47), as depicted in Figure 1.

Furthermore, several investigations have substantiated the impact of various viral infections both on sleep and immunity. Table 1 summarizes the studies highlighting the links among sleep, viral infections, and immunity.

**Table 1:** An overview on the reciprocal impact of specific viral infections on sleep parameters and innate immunity is summarized.

**Study**	**Viral infection considered**	**Effect on sleep**	**Effect on immunity**
Anderson et al. (48) Dale et al. (49)	Lethargic viral encephalitis	Primary hypersomnolence	Formed antibodies against antigens of their basal ganglia, infiltrated mononuclear cells, and interferon-α
Fang et al. (50) Nami (51) Mathews et al. (52)	Influenza virus (types A and B)	Enhanced non-rapid eye movement sleep (NREM) and decreased rapid eye movement sleep (REM), despite a body temperature decline	Affecting T cells, macrophages, and respiratory epithelium, in the absence of protective antibody, infiltrated mononuclear cells, and interferon-α
Drake et al. (16)	Type 23 rhinovirus	Decreased total sleep time, decreased consolidated sleep, and reduced sleep efficiency Aggravated sleep disordered breathing	Induced interleukin-25 in lung and upper airway epithelial cells, infiltrated mononuclear cells, and interferon-α
Shapiro (18)	Varicella-zoster virus	Hypersomnia, increased slow-wave sleep density	Neurotropic microglial changes, infiltrated mononuclear cells, and interferon-α
Phillips et al. (53) Prospero-Garcia et al. (54)	Feline immunodeficiency virus (FIV)	Alterations in sleep macrostructure including sleep fragmentation and displacement of slow- wave sleep	Infiltration of mononuclear cells into various cortical and subcortical brain regions, infiltrated mononuclear cells, and interferon-α
Darko et al. (55) Ferini-Strambi et al. (56)	Human immunodeficiency virus	Fewer REM sleep episodes and greater periods of wakes after sleep onset	Massive apoptosis of CD4^+^ T cell, infiltrated mononuclear cells, and interferon-α
McCall et al. (57) Ravenholt et al. (58) Steljes et al. (59) Dahan et al. (60)	Poliovirus	Obstructive and mixed sleep apnea–hypopnea syndrome, periodic limb movement disorder	Infiltrated mononuclear cells and interferon-α
Thevarajan et al. (19) Sariol et al. (61) Li et al. (62) Li et al. (63) Altena et al. (42) Gualano et al. (32) Sher (64) Casagrande et al. (35) Voitsidis et al. (31)	COVID-19	Anxiety-related insomnia, decreased total sleep time, poorer self-reported physical health linked with sleep disturbance, poor subjective sleep quality, stress–sleep link upon home confinement, a prevalence of sleep disturbances in up to 38% of pediatric health care workers, the link between sleep disturbances, poor mental health, and suicide	Increased antibody-secreting cells, follicular helper T cells, activated CD4^+^ T cells, and CD8^+^ T cells and immunoglobulin M (IgM) and IgG antibodies that bound the COVID-19–causing coronavirus SARS-CoV-2 (severe acute respiratory syndrome coronavirus 2)

Recently, in a review article by Ibarra-Coronado et al. (17), it is indicated that the disrupted sleep macrostructure in viral infections is proportionately linked with altered levels of proinflammatory cytokines, namely, interferon-α. In other words, sustained exposure to proinflammatory mediators and innate immune molecules, e.g., interferon-α, may modulate neuroinflammation and causes clinical symptoms of insomnia, arousal, and diminished sleep efficiency (17).

Anxiety can be related with mental impairment where exogenous and endogenous factors are involved in triggering neurological disorders (11, 23). Multiple lines of evidence support a relationship between sleep disorder produced by anxiety and its influence on the immunological system deterioration. A poor defense mechanism can reduce the possibility of a rapid response against viral infections.

**Figure 2:**
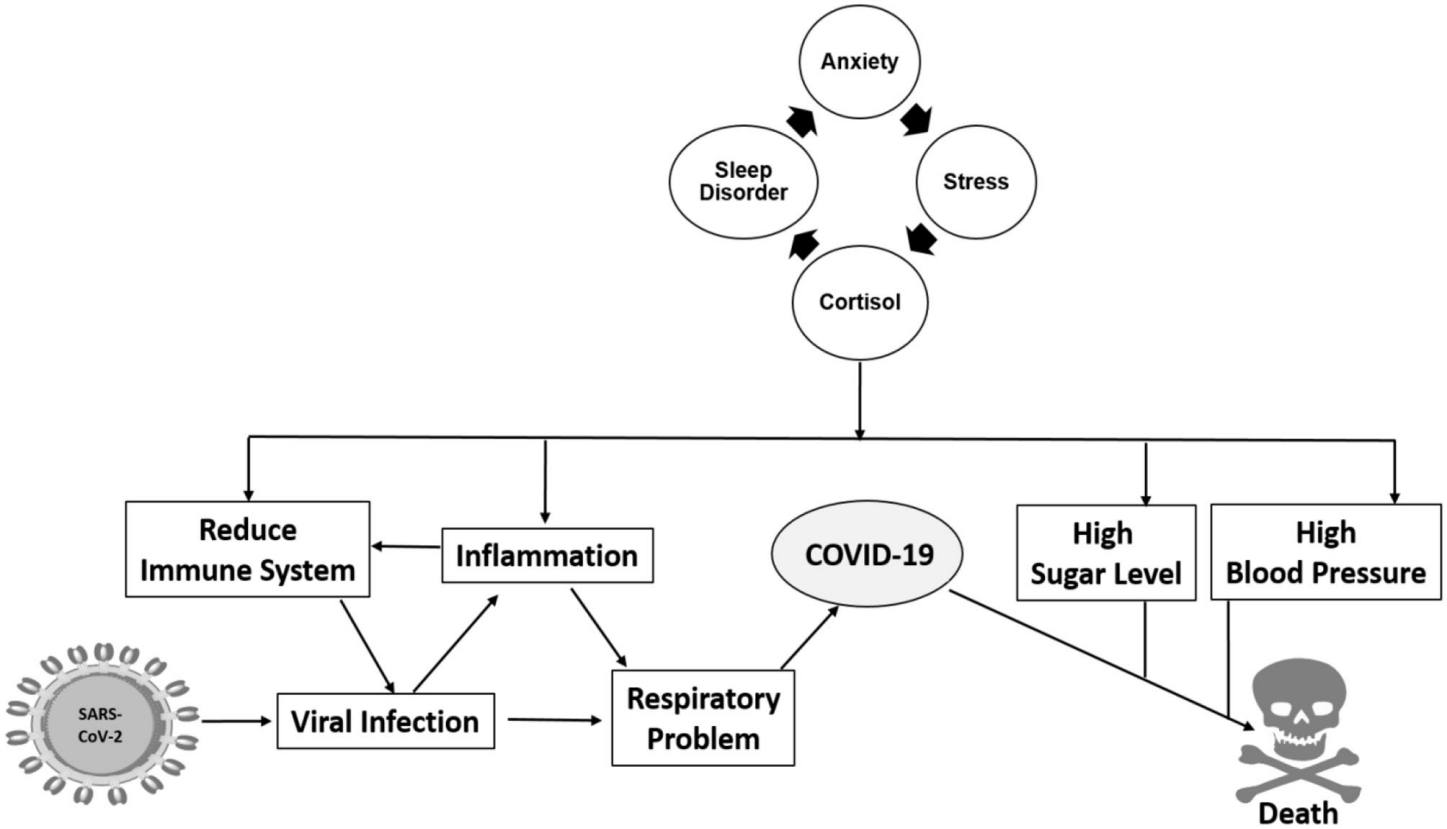
Sleep disorder is intrinsically related to anxiety which increases the possibility of viral infection through multiple pathways of inflammation, hypertension, high sugar levels, and others.

In Figure 2, we propose a relationship between sleep disorder with anxiety and high-risk factors that make humans susceptible to a viral infection, especially during this pandemic. Anxiety is now a common word used as a collateral effect of the COVID-19 pandemic situation, causing an increase in stress that causes cortisol hormone production (11, 23, 65). Cortisol is related to an inhibition of the immune system, which affects our ability of defending against viral and bacterial infections. Other conditions, such as inflammation, high sugar, and high blood pressure levels lessen human body health. Consequently, we consider that viral infections, particularly severe acute respiratory syndrome coronavirus 2 (SARS-CoV-2), can more strongly affect people with a sleep disorder. The infection caused by SARS-CoV-2 produces a hyperinflammation known as cytokine storm that affects antibody production, a very important defense mechanism. COVID-19 severely affects people with comorbidities, such as hypertension, diabetes, and cardiovascular disease (66, 67). These comorbidities produce inflammation that exacerbates during SARS-CoV-2 infection, causing an increase in mortality. Anxiety could be considered another potential comorbidity affecting COVID-19 patients, given that it also increases inflammation, high sugar level, and hypertension and produces cardiovascular disorder. Based on this, sleep disorders require more attention during this pandemic situation. We propose a direct relationship between sleep disorder with viral infection, where if sleep disorder is regulated, a decrease in SARS-CoV-2 related mortality rate may occur.

## Nutraceuticals in the Treatment of Anxiety

Because there is a reciprocal dynamic between anxiety and sleep disorders, it is crucial to find therapeutic strategies that tackle both issues without adverse effects. In this scenario, the use of nutraceuticals could be a practical, safe approach to regulate anxiety and sleep disorders. Moreover, a vast body of literature supports the use of nutraceuticals as an effective method to treat anxiety by regulating sleep disorders or *vice versa* (68). Therefore, we will further discuss the biological effects of the most recognized plant-based nutraceuticals, such as chamomile, valerian, cherries, kava kava, and caffeine, known for alleviating anxiety and sleep disorders.

*Matricaria chamomilla* L., commonly known as chamomile, is a dietary supplement that contains considerable amounts of bioactive flavonoids, such as apigenin and luteolin (69). Studies with isolated blood vessels from two different animal models demonstrate that apigenin and luteolin enhance vascular relaxation by activating nitric oxide (NO) signaling pathway and consequently regulating cyclic guanosine monophosphate (cGMP) levels (69, 70). The vascular relaxation caused by NO signaling activation induces smooth muscle relaxation, resulting in heart rate and blood pressure drop, which are essential processes during the non-REM sleeping stage (71). Furthermore, studies with cGMP-dependent protein kinase type I (PRKG1)–deficient mice showed aberrant sleep patterns, whereas cGMP-dependent PRKG2-deficient mice exhibited an anxiogenic behavioral profile (72, 73), thus suggesting that NO signaling plays an important role in sleep, circadian rhythm regulation, and anxiety processes. Overall, these data suggest the potential benefits of chamomile as a NO–cGMP signaling activator that favors sleep.

Another nutraceutical used to treat sleep disorders is valerian, a perennial herb with potent sedative properties (68, 74). Valerian increases the levels of γ-aminobutyric acid (GABA) in the brain, the primary inhibitory neurotransmitter in the central nervous system (CNS) that maintains the balance between neuronal excitation and inhibition (75). Valerenic acid, in particular, has a high binding affinity for the GABA_A_ receptor in neurons, similarly as the benzodiazepine class of tranquilizer drugs (75). GABA_A_ receptor activation promotes NREM sleep and significantly increases REM sleep (76). Recent studies also show that velerenic acid mitigates physical and psychological stress by reducing serum corticosterone levels and turnover of serotonin and norepinephrine, in addition to increasing BDNF levels in neuronal cells (77, 78). Therefore, valerian extract is a herbal supplement with proven anxiolytic and relaxation benefits.

Cherries and cherry juices are considered nutraceutical products with a diversity of health benefits, especially for sleep improvement. For instance, Jerte valley cherries have high concentrations of tryptophan, serotonin, and melatonin, which are neurochemicals with an important role in the physiological regulation of sleep (79). However, there are few reports on the beneficial effects of cherries in the improvement of sleep disorder (55).

Kava kava is another type of nutraceutical product with traditional and modern clinical use as a relaxant and anxiolytic treatment (80). Kavalactone is the main bioactive molecule present in kava kava, which exerts anxiolytic effects through various neurophysiological activities. For instance, the inhibition of voltage-dependent sodium channels reduced release of excitatory neurotransmitters by blocking calcium channels and potentiation of GABA_A_ receptor activity (81, 82). Furthermore, kavalactones reduce dopamine and noradrenaline's neuronal reuptake, which are crucial neuromodulators for maintaining important processes, such as wakefulness and vigilance (71). A recent meta-analysis of kava kava vs. placebo showed that three of seven clinical trials support that kava kava is more effective, suggesting that extracts from this root may work for treating anxiety in the short term (83).

Caffeine is considered a nutraceutical and pharmaceutical product that has different biochemical actions in the CNS. Among them is the up-regulation of hippocampal BDNF, a neurotrophic factor that is reduced during psychosocial stress, as previously mentioned (84). Moreover, moderate caffeine intake can counterbalance excessive sleepiness observed in people undergoing depressive states and improve mood states (85–87). A recent study by Wadhwa et al. found that caffeine alleviates sleep deprivation–induced inflammatory response and anxious behavior in rats by inhibiting microglia activation (88). Ultimately, caffeine can protect against alterations caused by anxiety and sleep disorders and potentially break the positive feedback loop between immune system alterations, anxiety, and sleep disorders.

In conclusion, including these types of nutraceutical products in the diet of people with sleep and anxiety disorders can help them to improve their mental health. Calming nutraceuticals could, therefore, regulate the excess or absence of sleepiness that considerably affects healthy lifestyle routines that are essential for developing a normal behavior. Natural treatment of anxiety and sleep disorders can be an alternative to boost the immune system, which is essential for an adequate response against viral infections, such as the one caused by SARS-CoV-2.

## Concluding Remarks; the Trilateral Interface

Based on the existing evidences (15–19, 48–60), there seem to be notable links among the immune system's aptitude, anxiety, and sleep efficiency. To favorably boost innate immunity, one of the health pillars is to have enough sleep and consider receiving proper treatment in case of sleep disorders. Anxiety is aggravated upon sleep loss, and sleep loss, in turn, results in aggravated anxiety. The collective effect of anxiety and sleep insufficiency would negatively affect the innate immunity against viral infections (the trilateral interface) including COVID-19.

It is proposed that results from current and future studies and related meta-analyses will form as guiding forces for investigations on the relationships between a stressful event and an immune parameter to understand the psychological phenomena that may mediate the relationship between sleep anxiety and immunity.

Further, the disease is characterized by natural or specific immunity, its cytokine profile, and its regulation by anti-inflammatory agents, such as cortisol, which may determine the disparate effects of different kinds of stressors including inefficient sleep on individual's adaptive immunity profile.

Because it might be difficult for many people these days to properly adhere to sleep hygiene measures and mental health advice, it might be worthwhile to develop online consultation platforms for cognitive–behavioral therapies and educational webinars. This would help individuals conquer their sleep insufficiency, anxiety, and hampered immunity against viral infections.

Despite the available evidence, it seems that the current state of knowledge on anxiety, sleep, and COVID-19 is still based on preexisting research, and further investigations are still needed to explore the relationship among these variables providing new dimensions in understanding the complex nature of mental health.

## Author Contributions

All authors listed have made a substantial, direct and intellectual contribution to the work, and approved it for publication.

## Conflict of Interest

The authors declare that the research was conducted in the absence of any commercial or financial relationships that could be construed as a potential conflict of interest.
